# The 2‐oxoglutarate/malate carrier extends the family of mitochondrial carriers capable of fatty acid and 2,4‐dinitrophenol‐activated proton transport

**DOI:** 10.1111/apha.14143

**Published:** 2024-04-05

**Authors:** Kristina Žuna, Tatyana Tyschuk, Taraneh Beikbaghban, Felix Sternberg, Jürgen Kreiter, Elena E. Pohl

**Affiliations:** ^1^ Physiology and Biophysics, Department of Biological Sciences and Pathobiology University of Veterinary Medicine Vienna Austria; ^2^ Present address: Ludwig Boltzmann Institute for Traumatology, The Research Centre in Cooperation with AUVA Vienna Austria; ^3^ Present address: Institute of Molecular and Cellular Physiology Stanford University School of Medicine Stanford California USA

**Keywords:** long‐chain fatty acids, mitochondrial transport, planar bilayer membranes, proton transport, SLC25A11, total membrane conductance

## Abstract

**Aims:**

Metabolic reprogramming in cancer cells has been linked to mitochondrial dysfunction. The mitochondrial 2‐oxoglutarate/malate carrier (OGC) has been suggested as a potential target for preventing cancer progression. Although OGC is involved in the malate/aspartate shuttle, its exact role in cancer metabolism remains unclear. We aimed to investigate whether OGC may contribute to the alteration of mitochondrial inner membrane potential by transporting protons.

**Methods:**

The expression of OGC in mouse tissues and cancer cells was investigated by PCR and Western blot analysis. The proton transport function of recombinant murine OGC was evaluated by measuring the membrane conductance (*G*_m_) of planar lipid bilayers. OGC‐mediated substrate transport was measured in proteoliposomes using ^14^C‐malate.

**Results:**

OGC increases proton *G*_m_ only in the presence of natural (long‐chain fatty acids, FA) or chemical (2,4‐dinitrophenol) protonophores. The increase in OGC activity directly correlates with the increase in the number of unsaturated bonds of the FA. OGC substrates and inhibitors compete with FA for the same protein binding site. Arginine 90 was identified as a critical amino acid for the binding of FA, ATP, 2‐oxoglutarate, and malate, which is a first step towards understanding the OGC‐mediated proton transport mechanism.

**Conclusion:**

OGC extends the family of mitochondrial transporters with dual function: (i) metabolite transport and (ii) proton transport facilitated in the presence of protonophores. Elucidating the contribution of OGC to uncoupling may be essential for the design of targeted drugs for the treatment of cancer and other metabolic diseases.

## 1 INTRODUCTION

Cancer cells demonstrate distinctive metabolic reprogramming, relying more on aerobic glycolysis than oxidative phosphorylation (OXPHOS) for energy production, and exhibiting increased glutaminolysis and oxidative stress.^1^, ^2^ In particular, glutamine‐derived α‐ketoglutarate (α‐KG), also known as 2‐oxoglutarate, can participate in the OXPHOS pathway or the reductive carboxylation pathway, deviating from the usual TCA cycle. The 2‐oxoglutarate/malate carrier (OGC) aids this by transporting 2‐oxoglutarate and malate across the mitochondrial membrane.^2^ Recent evidence shows that knockdown of OGC results in a 75% reduction in ATP production in non‐small cell lung cancer.^3^ In addition, a heterozygous OGC knockout reduced the growth of spontaneous lung cancer in mice by 50%, while inhibition of OGC by N‐phenylmaleimide resulted in a 50% reduction in melanoma formation in human xenograft models.^4^ The inhibitory effect on malignant growth has been attributed to the involvement of OGC in the replenishment of NADH required for ATP production via the malate–aspartate shuttle. Neutralization of excessive amounts of reactive oxygen species (ROS) by increasing proton transport across the membrane may be an alternative strategy and has been shown to induce apoptosis in mature tumors or prevent damage early in cancer development and after radiation therapy.^5^ Application of chemical protonophores such as BAM15, niclosamide, or oxyclozanide to in vitro and in vivo systems resulted in a significant reduction in tumor proliferation.^6^, ^7^, ^8^

OGC, a member of the mitochondrial solute carrier 25 (SLC25) superfamily, is located in the inner mitochondrial membrane (IMM), where it transports 2‐oxoglutarate for L‐malate or other C4 metabolites.^9^ Notably, the double knockout of OGC is embryonically lethal.^3^ OGC was previously used as a negative control for uncoupling^10^, ^11^ until Yu et al. observed a significant decrease in mitochondrial membrane potential (MMP) after its overexpression in HEK293 cells.^12^ Later, glutathione transport by OGC was proposed to play a role in reducing of oxidative stress in neuronal cells,^13^ but this function remained controversial.^14^ Since oxidative stress is reduced by mitochondrial uncoupling,^15^, ^16^ the real reason may be OGC‐mediated H^+^ transport, which has never been confirmed and further investigated in a well‐defined system.

Since the homologs of OGC—adenine nucleotide transporter 1 (ANT1, 28% homology to OGC) and uncoupling proteins 1 (UCP1, 33% homology to OGC)—enhance H^+^ transport in the presence of free fatty acids (FAs) and 2,4‐dinitrophenol (DNP)^17^, ^18^ we hypothesized that also OGC dissipates the IMM proton gradient under similar conditions. Therefore, in this work, we used a well‐defined system of planar lipid bilayer membranes reconstituted with OGC to (i) investigate its contribution to FA‐ and DNP‐mediated proton transport, (ii) evaluate the efficiency of OGC inhibitors in reducing the proton transport rate, and (iii) identify amino acids critical for the interaction of OGC with FAs.

Unraveling the physiological function and mechanism of OGC‐mediated mitochondrial uncoupling has the potential to provide insight into its involvement in cancer metabolism and aid in the development of targeted drugs for other metabolic diseases.

## 2 RESULTS

### 2.1 OGC is present in a wide range of murine tissues and cancer cell lines

**FIGURE 1:**
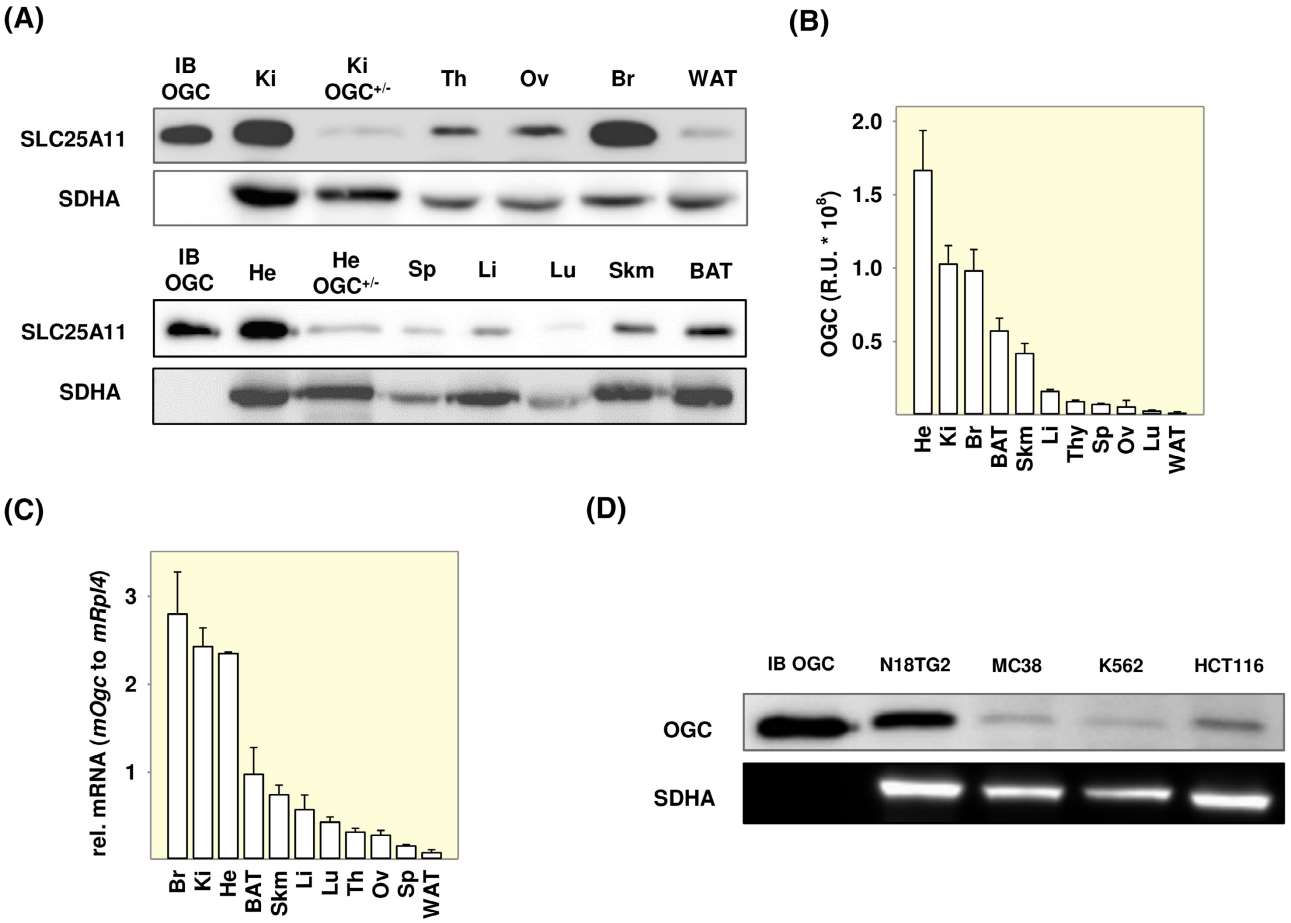
Detection of OGC (SLC25A11) in mice tissues and cancer cell lines. (A) Representative Western blot of OGC in mouse tissues. Tissues are labeled as follows: kidney (Ki), thymus (Th), ovary (Ov), brain (Br), white adipose tissue (WAT), heart (He), spleen (Sp), liver (Li), lung (Lu), skeletal muscle (Skm), and brown adipose tissue (BAT). (B) Quantification of OGC band intensity in all tissue samples in relative units. (C) mOGC mRNA levels (2‐ΔCt) relative to mitochondrial ribosomal protein L4 (mRPL4) used as a reference gene. (D) Western blot analysis of OGC in mouse neuroblastoma (N18TG2), mouse colon adenocarcinoma (MC38), human myeloid leukemia (K562), and human colorectal carcinoma (HCT116) cell lines. 40 μg (A) or 20 μg (D) of total protein was loaded per lane. Recombinant mouse OGC (mOGC) inclusion bodies (IBs) (IB OGC, 2 μg) were used as positive controls, and mOGC heterozygous heart (He+/−) and kidney (Ki+/−) knockouts as negative controls in (A). OGC is detected at the corresponding size of 34 kDa. Succinate dehydrogenase (SDHA) was used as a mitochondrial control. Reversible Ponceau S staining loading control, full blots, and anti‐OGC antibody validation are shown in Figure S1. See Section “4” for more details.

To understand the physiological role of OGC, we first investigated its tissue expression in mice. Based on immunohistochemical staining experiments and testing the mRNA abundance, OGC has been reported to be ubiquitously expressed in the human body,^19^ and in various rat tissues.^20^ However, mRNA levels are not reliable predictors of protein presence^21^ and there are no experimental data on OGC protein expression in mice. The main problem in testing the expression of SLC25 superfamily proteins is the lack of specific antibodies capable of distinguishing close homologs. To address this issue, we first validated the anti‐OGC (anti‐SLC25A11) antibody using inclusion bodies (IBs) of mouse OGC (mOGC) and other SLC25 family members, as well as heterozygous mouse knockout tissues. As shown in Figure S1, the antibody was specifically bound only to OGC IBs. We found that OGC was expressed on the protein level in all tested murine tissues (Figure 1A,B), with an expected decrease in heterozygous knockout samples. OGC was also present at the mRNA level in all tissues (Figure 1C), and in both cases, the highest expression was found in the heart, brain, and kidney. In addition, we have shown that OGC is expressed at different levels in murine and human cancer cell lines (Figure 1D).

### 2.2 Production and reconstitution of mouse OGC in liposomes

**FIGURE 2:**
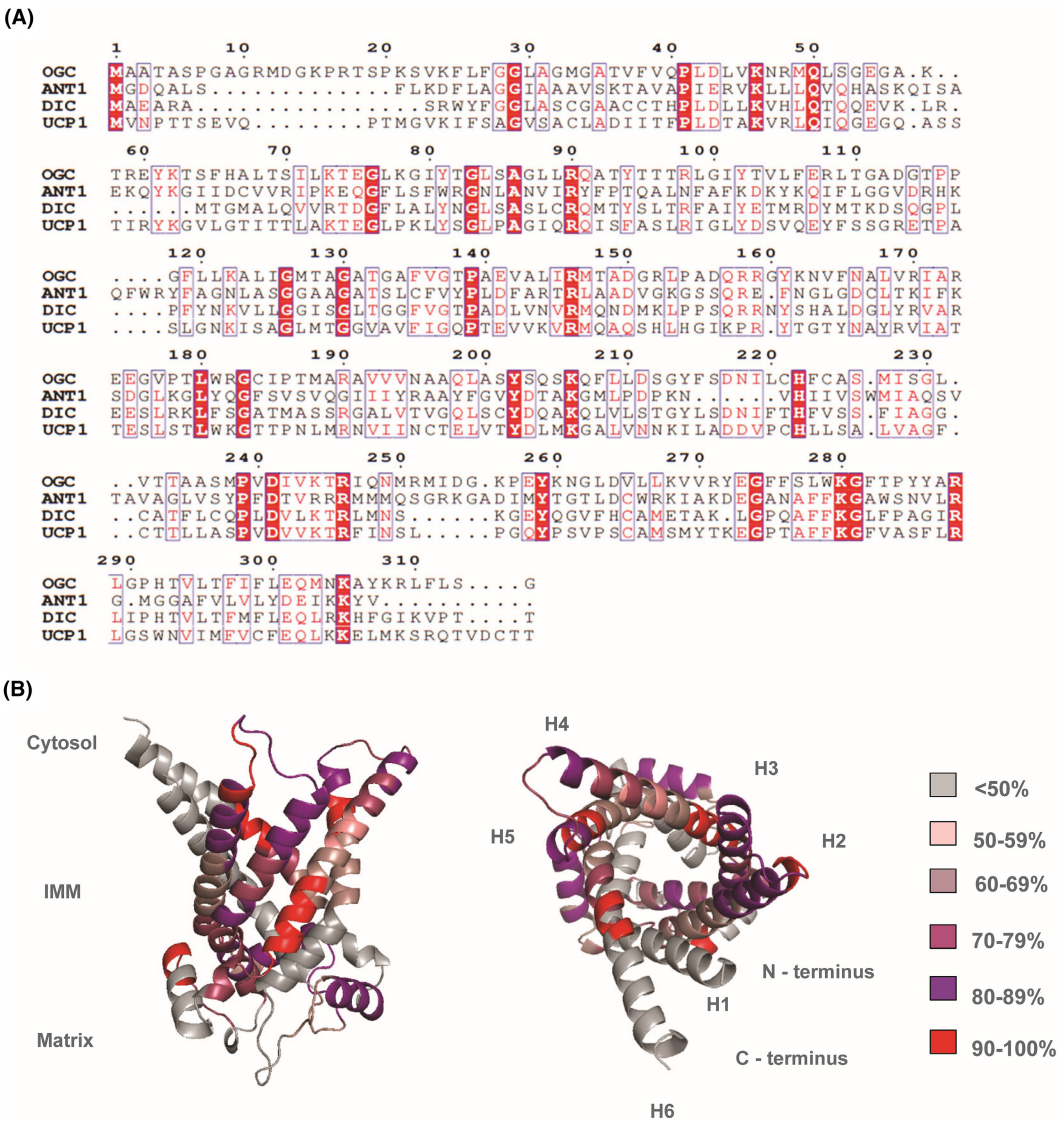
High sequence homology in substrate binding sites and signature motifs between OGC and other members of the SLC25 family. (A) Sequence alignment of murine OGC, adenine nucleotide translocase 1 (ANT1), dicarboxylate carrier (DIC), and uncoupling protein 1 (UCP1) generated with ClustalW and colored with ESPript. (B) Sequence homology scores between mOGC and mANT1. Homology scores are averaged for every 5 amino acids, based on their individual homology percentage scores calculated in the alignment. The image was generated in PyMOL using the AlphaFold structure of mOGC (AF‐Q9CR62‐F1) as a template. Alpha helices are numbered as H1–H6. First 20 amino acids of the N‐terminus are not shown for simplicity.

OGC was the first eukaryotic protein to be expressed in Escherichia *coli* (*E. coli*) and functionally reconstituted in proteoliposomes.^9^ The homology between OGC and other proteins of the SLC25 superfamily, such as ANT1, UCP1, and the dicarboxylate carrier is high, especially in conserved mitochondrial carrier motifs and substrate binding sites (Figure 2). Therefore, it is possible to express and purify them by using similar protocols.

**FIGURE 3:**
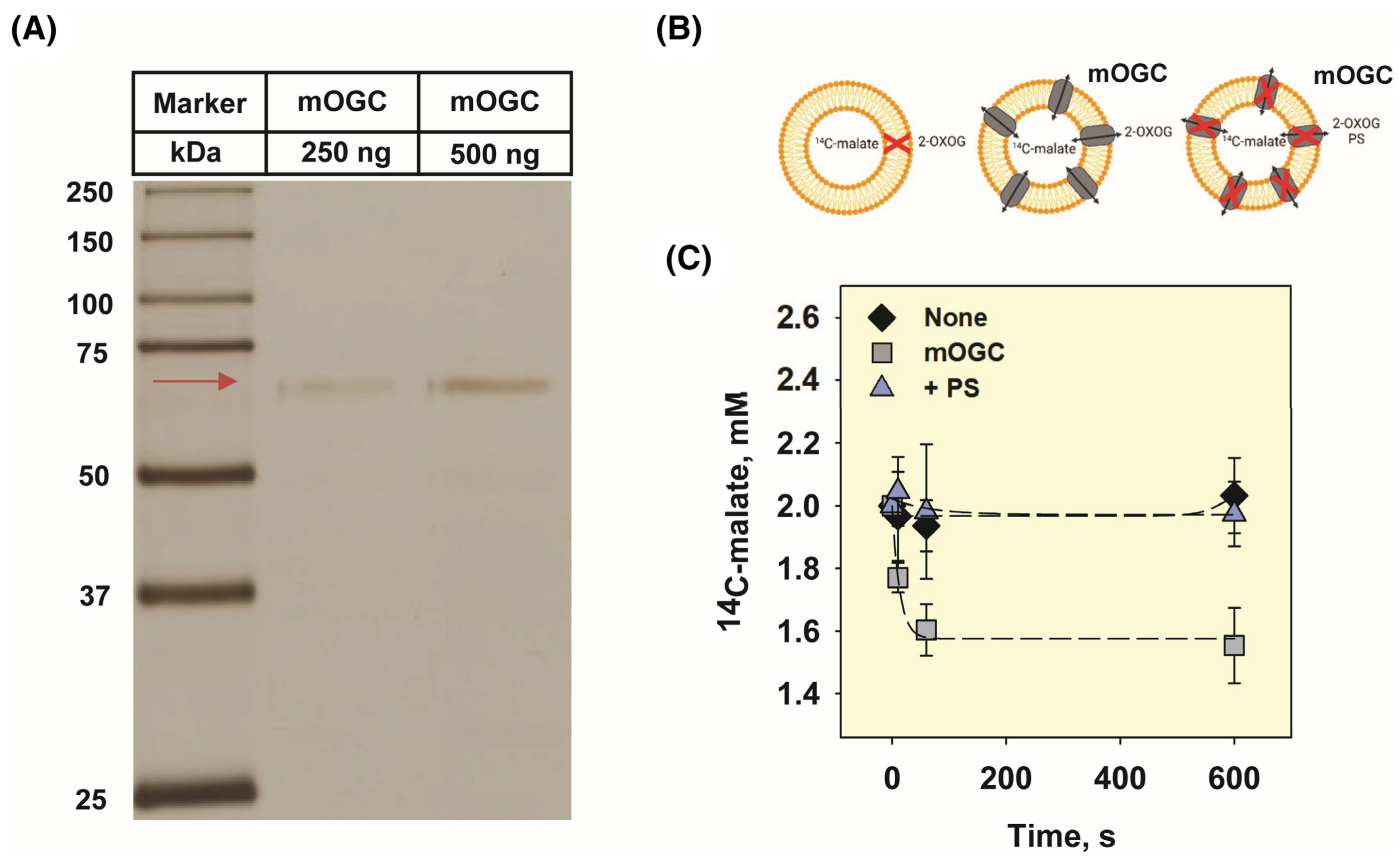
Reconstituted mOGC transports 14C‐malate for 2‐oxoglutarate. (A) Representative silver staining of mOGC reconstituted into proteoliposomes. 250 and 500 ng of proteoliposomes were loaded in separate lanes on a 15% acrylamide gel, separated by SDS‐PAGE and visualized by silver staining. Reconstituted mOGC appears at the expected dimer size (~69 kDa, red arrow). Precision Plus Protein Dual Color Standard was loaded as a molecular weight marker. (B) The scheme shows the three experimental setups: empty liposomes (left), active transport with mOGC (middle), and inhibition of transport by phenylsuccinate (PS, right). (C) Decrease in 14C‐malate concentration upon initiation of 2‐oxoglutarate/malate exchange in proteoliposomes containing reconstituted mOGC (squares). Transport was not measured in empty liposomes (diamonds) and was completely inhibited by the addition of 20 mM of the substrate analog PS (triangles). For all measurements, membranes were prepared DOPC:DOPE:CL (45:45:10 mol%). Lipid and protein concentrations were 1.5 mg/mL and 4 μg/(mg lipid), respectively. The buffer solution consisted of 50 mM Na_2_SO_4_, 10 mM Tris, 10 mM MES, and 0.6 mM EGTA at pH 7.34 and *T* = 32°C. Hundred nanometer proteoliposomes were filled with 2 mM 14C‐malate and transport was initiated by the addition of 2 mM 2‐oxoglutarate from the outside. 14C‐malate, oxoglutarate, and PS were dissolved in buffer (pH 7.34). Data are mean ± SD of at least three independent experiments.

In this study, we adapted previously established protocols to produce murine OGC (mOGC) in *E. coli* IBs.^9^, ^17^, ^22^ After purification and reconstitution into proteoliposomes (see Section 4), it was present as a dimer (Figure 3A). Early cross‐linking studies suggested possible cysteine‐linked dimerization of OGC in detergent,^23^ although the issue remained controversial,^24^ and the observed dimerization may be a result of aggregation under SDS‐PAGE conditions.^25^, ^26^

### 2.3 mOGC transports ^14^C‐malate for 2‐oxoglutarate

To confirm the functionality of mOGC and its correct refolding in proteoliposomes, we performed substrate transport exchange measurements using ^14^C‐malate and 2‐oxoglutarate (Figure 3B). Figure 3C shows the decrease in ^14^C‐malate concentration in proteoliposomes reconstituted with mOGC upon the addition of 2‐oxoglutarate. The transport rate, *τ*, was estimated from the decrease in ^14^C‐malate concentration over time, normalized to the protein concentration. The determined τ was approximately 47.24 μmol/min/mg, which is in good agreement with previous results for mitochondrial carriers (Table S1). Transport was completely inhibited by phenylsuccinate (PS), a substrate analog and a known inhibitor of the transport function of OGC.^27^

### 2.4 Fatty acid and 2,4‐dinitrophenol‐mediated proton transport is facilitated by mOGC in planar lipid bilayers

**FIGURE 4:**
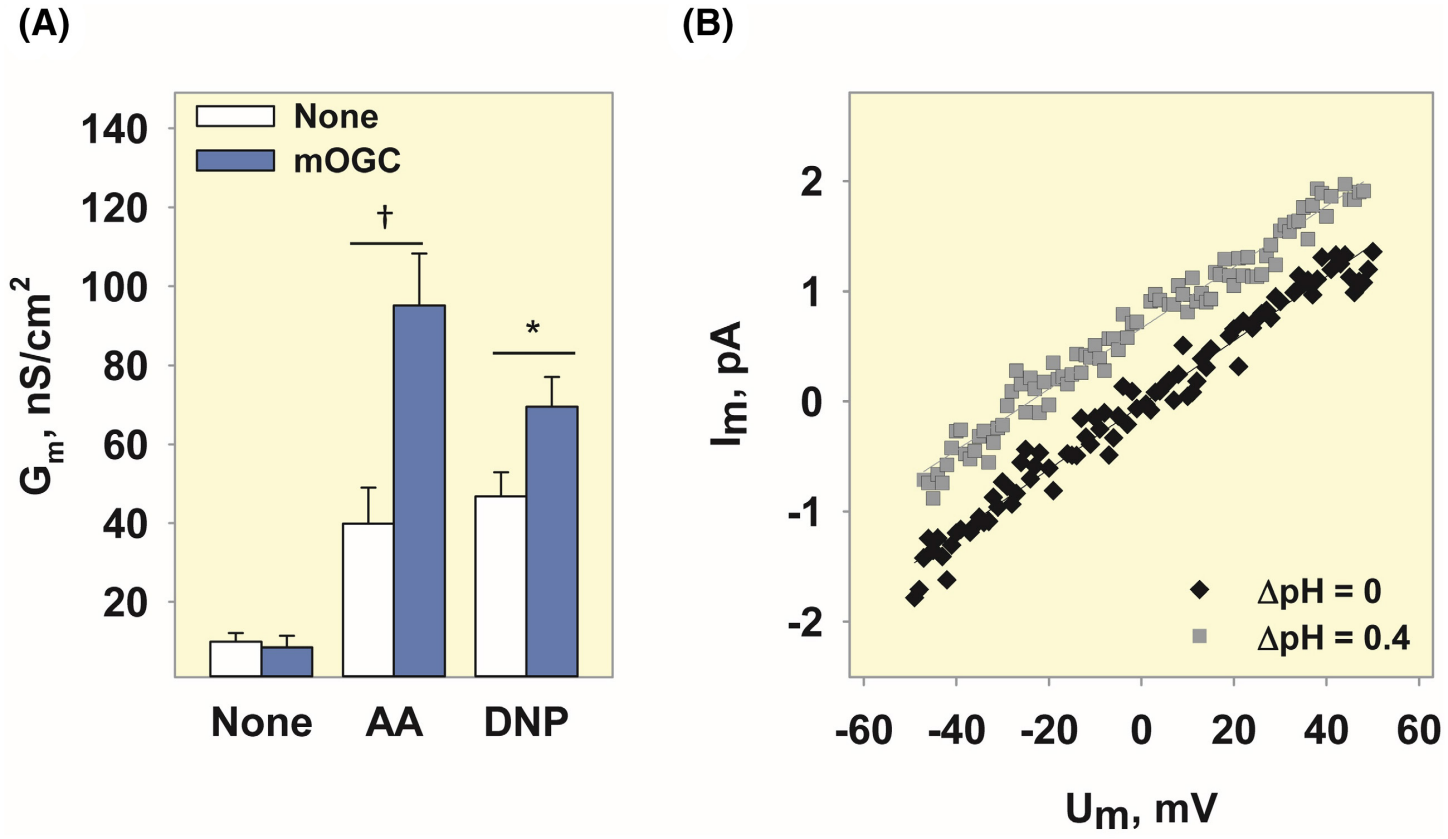
mOGC‐mediated proton conductance in the presence of AA or DNP in planar lipid bilayers. (A) Increase in total membrane conductance (*G*_m_) in the presence of 15 mol% arachidonic acid (AA) or 50 μM DNP without (white) and with (blue) mOGC. (B) Current–voltage (I/V) recordings of lipid bilayer membranes reconstituted with mOGC in the presence and absence of a transmembrane pH gradient of 0.4 pH units. For all measurements, membranes were made prepared from DOPC:DOPE:CL (45:45:10 mol%). Lipid and protein concentrations were 1.5 mg/mL and 4 μg/(mg lipid), respectively. The buffer solution consisted of 50 mM Na_2_SO_4_, 10 mM Tris, 10 mM MES, and 0.6 mM EGTA at pH 7.34 and *T* = 32°C. DNP and ATP were dissolved in DMSO and buffer (pH 7.34), respectively. Data are mean ± SD of at least three independent experiments.

Next, we tested whether mOGC contributes to the proton conductance in pure lipid membranes composed of DOPC, DOPE, and cardiolipin (45:45:10 mol%) in the model system.^28^ We first measured the increase in the total membrane conductance (*G*_m_) in the presence of a representative natural protonophore, arachidonic acid (AA), and DNP, a very potent chemical uncoupler that was previously used for obesity treatment.^29^ *G*_m_ of protein‐free planar lipid bilayers (*G*_m_ = 8.75 ± 3.2 nS/cm^2^) is comparable to the conductance of membranes reconstituted with mOGC alone (*G*_m_ = 9.23 ± 3 nS/cm^2^). The *G*_m_ of bilayers containing AA (*G*_m_ = 34.1 ± 7.1 nS/cm^2^) or DNP (*G*_m_ = 46.8 ± 6.1 nS/cm^2^) was significantly increased in the presence of mOGC (*G*_m_ = 92.5 ± 16.5 nS/cm^2^ for AA, *G*_m_ = 69.6 ± 7.5 nS/cm^2^ for DNP; Figure 4A and Figure S2).

To confirm that in the presence of AA, mOGC facilitates the transport of protons, and not other ions present in the buffer, we measured current–voltage characteristics in the presence and absence of a transmembrane pH gradient of 0.4 (ΔpH 0.4) (Figure 4B). Solutions on both the *cis* and *trans* sides of the planar lipid bilayer had the same concentrations of all ions except H^+^ and OH^−^, similar ionic strength, and similar osmolarity. The pH values on the *cis* and *trans* sides were 7.34 and 7.74, the latter being adjusted with 2.39 mM of Tris dissolved in water (pH 7.34) after bilayer membrane formation. In this case, the experimentally obtained shift of the reversal potential, ΔV_0_, is equal to the theoretical H^+^ Nernst potential (Ψ_N_) at ΔpH 0.4. The shift of intersection points of *I*/*V* curves with the *x*‐axis, for ΔpH 0 and ΔpH 0.4, resulted in *V*_0_ = 25.8 ± 6.8 mV (Figure 4B). We then calculated the transfer number of H^+^ and OH^−^ ions across the membrane (Equation 1),
(1)

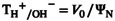

where *Ψ*_N_ is the theoretical value of H^+^ Nernst potential at ΔpH 0.4 (23.9 mV). T_H_^+^_/OH_^−^ was 1.08 ± 0.3, which confirms that the observed increase in *G*_m_ is exclusively due to the transport of protons.

### 2.5 mOGC mediates proton transport in the presence of free fatty acid with different structures

**FIGURE 5:**
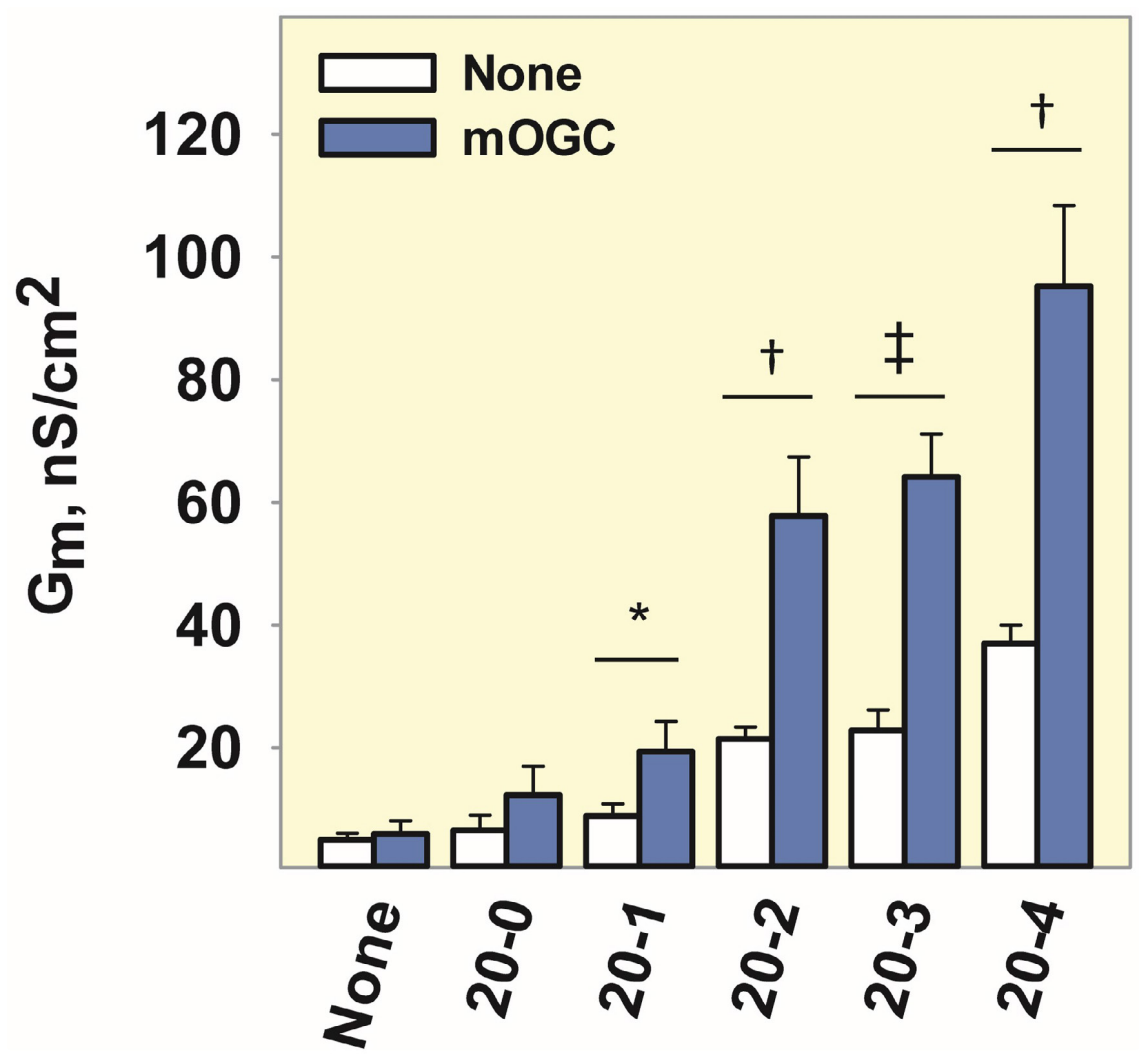
The increase in mOGC‐mediated proton transport directly correlates with the increase in the number of unsaturated bonds of FAs. Increase in *G*_m_ in the presence of arachidic acid (20‐0), cis‐11‐eicosaenoic acid (20‐1), cis‐11,14‐eicosadienoic acid (20‐2), cis‐8,11,14‐trienoic acid (20‐3), and AA (20‐4). The experimental conditions were similar to those described in Figure 4.

Under oxidative stress, the cell concentration of ROS increases and subsequently activates phospholipase A (PLA).^30^ PLA_2_ cleaves unsaturated FAs from the IMM, the most abundant of which is AA. Therefore, to understand how OGC‐mediated proton transport correlates with the degree of FA saturation as a response to oxidative stress in cells, we measured the *G*_m_ of mOGC‐reconstituted lipid bilayers in the presence of FAs with increasing number of double bonds—arachidic acid (20–0), cis‐11‐eicosaenoic acid (20–1), cis‐11,14‐eicosadienoic acid (20–2), cis‐8,11,14‐trienoic acid (20–3), and AA (20–4) (Figure 5). Our results show that the *G*_m_ is directly correlated with the increase in the number of unsaturated bonds of the FA, similar to what was observed for UCP1, UCP2, and ANT1.^31^, ^32^

### 2.6 The activation of mOGC by arachidonic acid can be inhibited by ATP, its substrates, and substrate analog

**FIGURE 6:**
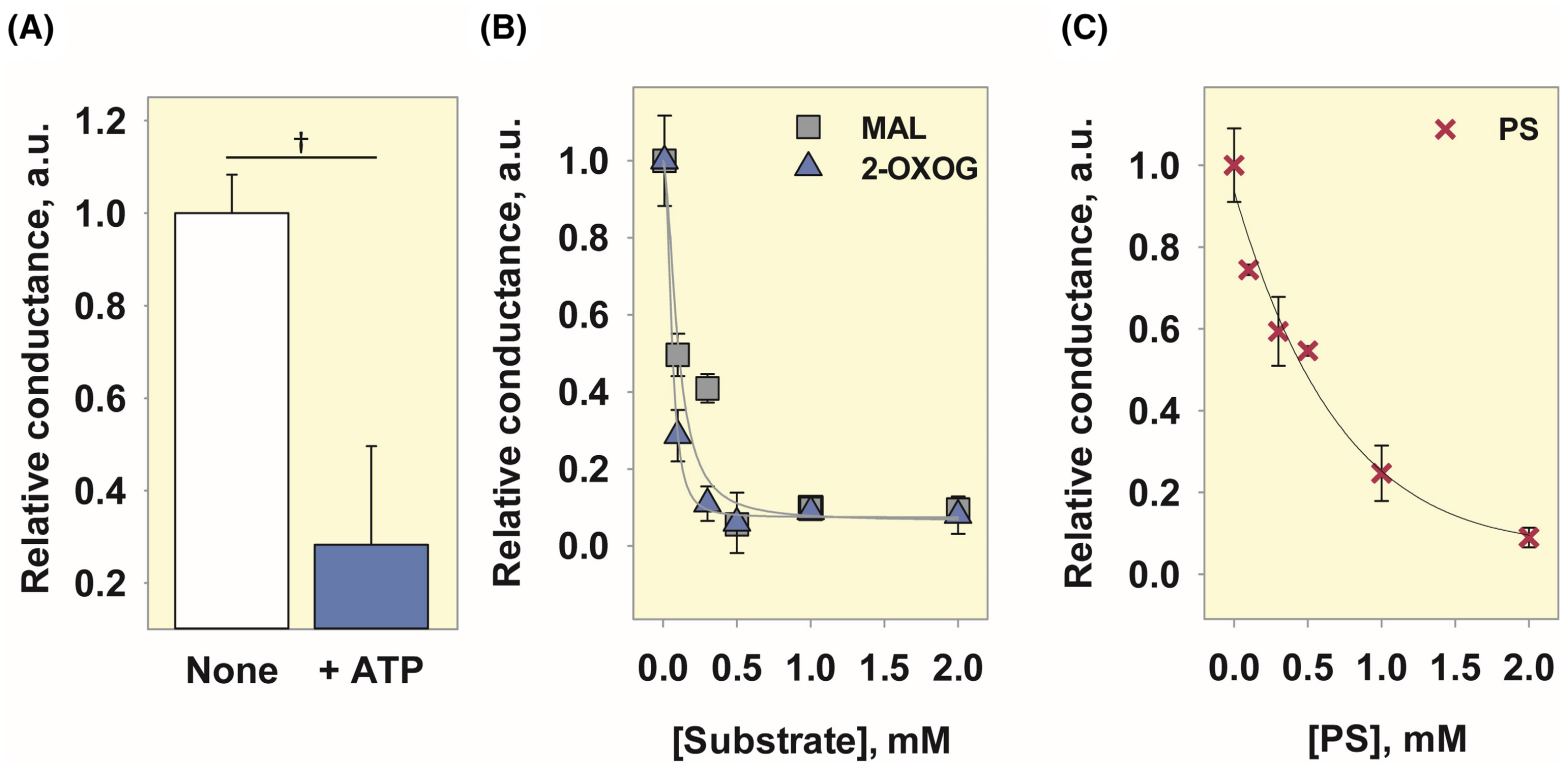
Inhibition of mOGC‐mediated proton transport in the presence of AA by ATP, 2‐oxoglutarate (2‐OXOG), malate (MAL) and phenylsuccinate (PS). Relative conductance of mOGC activated by 15 mol% AA and inhibited by 4 mM ATP (A), 2‐OXOG or MAL (B), or PS (C). The relative conductance is the ratio of the total membrane conductance in the presence and absence of the protein inhibitors to the membrane conductance measured in the presence of lipid and AA (see Section 4). The curves in (B) and (C) were fitted with a 4‐parameter sigmoidal function (least squares method) and IC50 values of (0.06 ± 0.01) mM for 2‐OXOG, (0.1 ± 0.06) mM for MAL and (1 ± 0.11) mM for PS were obtained. ATP, 2‐OXOG, and MAL were dissolved in buffer (pH 7.34). PS was dissolved in DMSO. Other experimental conditions were similar to those described in Figure 4.

We further tested whether ATP could inhibit mOGC‐enhanced proton transport in the presence of AA. Figure 6A and Figure S3A show that ATP can inhibit mOGC‐mediated proton conductance by 75%, indicating a possible common binding site for ATP and AA.

Next, we tested whether the main substrates transported by OGC, 2‐oxoglutarate, and malate, show a similar effect. Figure 6B and Figure S3B show that the rate of AA‐mediated proton transport inhibition in the presence of mOGC depends on the substrate concentration with IC50s of (0.06 ± 0.01) mM and (0.1 ± 0.06) mM for 2‐oxoglutarate and malate, respectively. This is consistent with the substrate transport function of OGC, and its highest affinity for 2‐oxoglutarate.^9^ We then found that the IC50 of PS was (1 ± 0.11) mM (Figure 6C, Figure S3C), higher than both substrates tested.

None of the tested compounds altered the *G*_m_ values when only mOGC or AA were present in the system (Figure S3D), confirming that there is no unspecific proton leak. Taken together, these results may indicate a common binding site, or a shared part of the binding site in OGC for AA, ATP, 2‐oxoglutarate, malate, and PS.

### 2.7 R90 is involved in the binding of free fatty acids, ATP, and substrates

**FIGURE 7:**
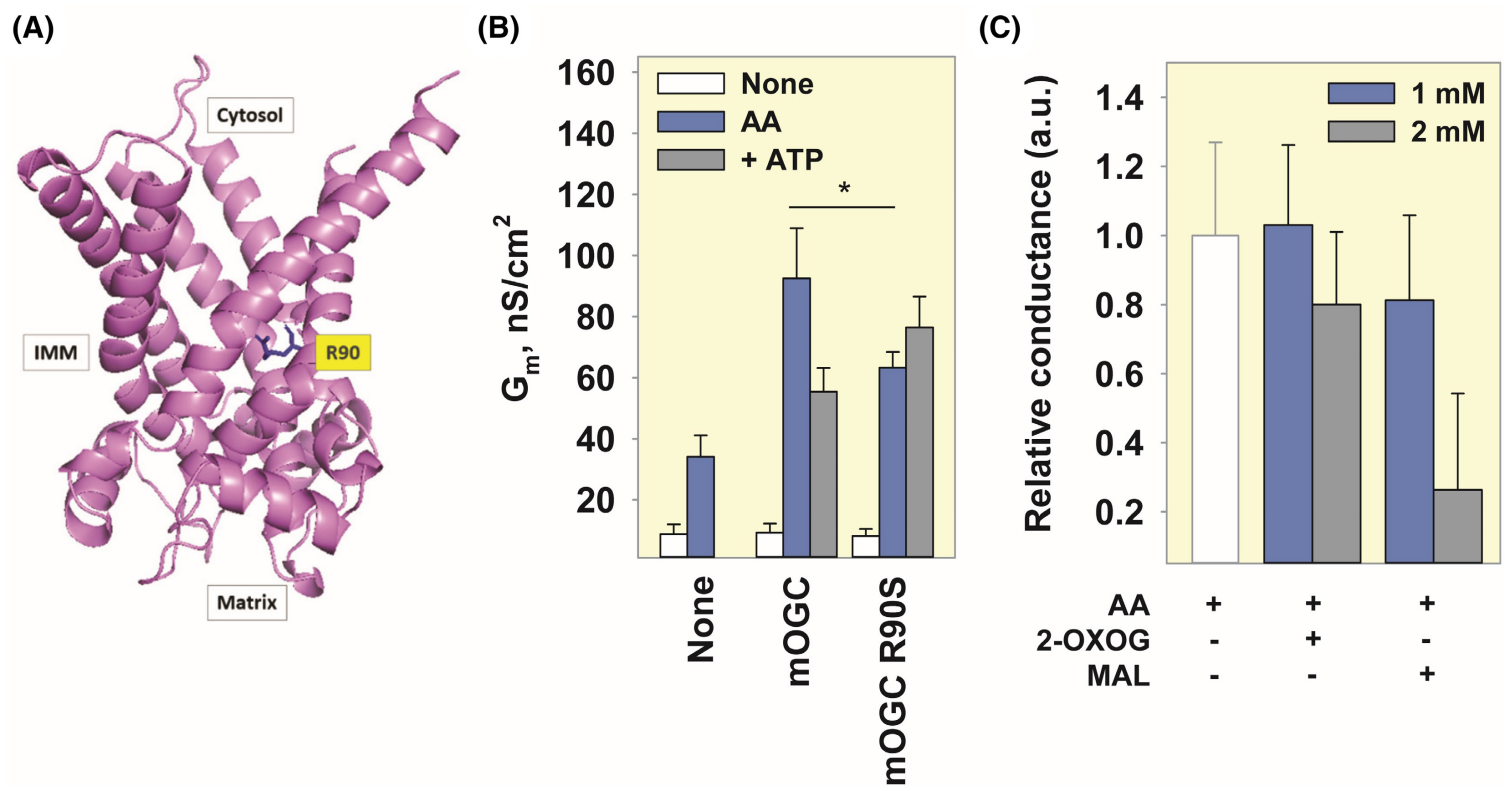
Substrate‐binding residue R90 is involved in uncoupling. (A) Side view of mOGC with R90 shown in blue licorice and labeled in yellow. The image was generated in PyMOL using the AlphaFold structure of mOGC (AF‐Q9CR62‐F1) as a template. The N‐terminal sequence has been truncated for simplicity. (B) Comparison of total membrane conductance (*G*_m_) between mOGC and mOGC R90S in the presence of 15 mol% AA (blue) or 4 mM ATP (gray). C. Relative *G*_m_ measured in the presence of different concentrations of 2‐OXOG or MAL. Other experimental conditions were similar to those described in Figure 4.

Residues R90, Y94, R98, R190, and R288 were identified as critical for substrate transport of OGC by substitution with cysteine and leucine.^33^, ^34^, ^35^ Using site‐directed mutagenesis, we produced mOGC‐R90S (Figure 7A) and compared the specific membrane conductance of the bilayer membranes reconstituted with the wild‐type and mutant proteins. Figure 7B shows that *G*_m_ is 50% lower in the presence of mOGC‐R90S (63.28 ± 5.12 nS/cm^2^ versus 92.47 ± 16.49 nS/cm^2^ measured for mOGC, relative to the AA‐induced *G*_m_). Furthermore, mutation of R90 to serine completely abolished the ability of ATP to inhibit mOGC. We also tested how efficiently the AA‐induced *G*_m_ of mOGC‐R90S can be inhibited by malate and 2‐oxoglutarate. Figure 7C and Figure S4 show that 1 mM of 2‐oxoglutarate or malate inhibited the activated mOGC by 0% and 20%, respectively, whereas 2 mM of 2‐oxoglutarate or malate inhibited the protein by 19% and 74%, respectively. These results suggest that R90 may be more important in the binding of 2‐oxoglutarate than malate in the protein's cavity, or that malate competes more efficiently with AA for the rest of the binding site.

## 3 DISCUSSION

**FIGURE 8:**
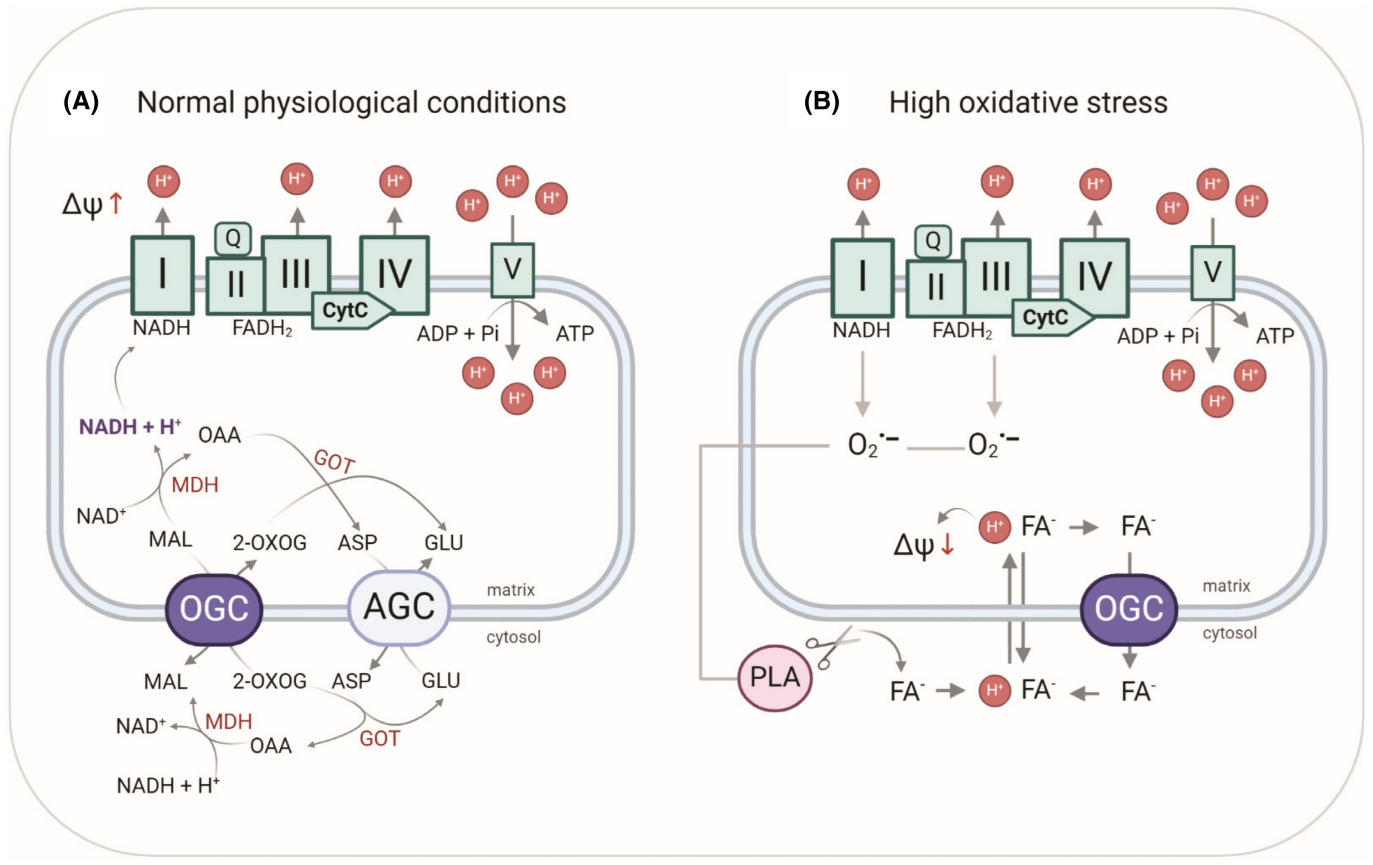
OGC catalyzes substrate transport and participates in FA‐mediated uncoupling. (A) Under physiological conditions, OGC transports 2‐oxoglutarate for malate to support the regeneration of NADH for oxidative phosphorylation (OXPHOS). The enzymes are the electron chain complex (I–IV), ATP synthase (V), aspartate/glutamate carrier (AGC), malate dehydrogenase (MDH), and glutamate oxaloacetate transaminase (GOT), and the substrates are malate (MAL), 2‐oxoglutarate (2‐OXOG), aspartate (ASP), glutamate (GLU), and oxaloacetate (OAA). (B) Under high oxidative stress, OGC is involved in FA‐mediated uncoupling. Leakage of electrons from electron transport complexes I and III generates reactive oxygen species (ROS), which in turn activate phospholipase A (PLA), which cleaves FAs from the IMM. After neutralization and transfer to the matrix, FAs release protons and decrease MMP. FA anions are transported back into the cytosol by OGC and similar proteins. Image is created in BioRender.

Our results show that OGC can participate in uncoupling under similar conditions as ANT1 and UCP1‐3 and that proton transport is enhanced only in the presence of the artificial uncoupler DNP or long‐chain FAs.^17^ Proton transport facilitated by OGC increased with the degree of unsaturation of the FA, as has been shown for other carriers.^31^, ^32^ Furthermore, ATP can competitively inhibit the activity of ANT1, UCP1‐UCP3, and OGC in the presence of AA, suggesting a common binding site for nucleotides. We identified R90 as one of the residues involved in the binding of AA and ATP to OGC (Figure 7). This amino acid is a part of the conserved common substrate binding site of the SLC25 family and is a homolog of R79 in ANT1 and R84 in UCP1, which are critical for the binding of nucleotides, FAs, and DNP.^32^, ^36^, ^37^, ^38^, ^39^, ^40^ It also has a homolog in UCP2 (R88) and dicarboxylate carrier (R69). Since these proteins are involved in both substrate transport and FA‐mediated proton transport, we propose that OGC is a protein with dual function whose substrate binding site is part of the interaction site with negatively charged uncouplers such as FAs or DNPs (Figure 8).

A possible explanation for thermogenesis‐independent uncoupling would be mild uncoupling or lowering of MMP to prevent the generation of damaging ROS.^41^, ^42^ Under normal physiological conditions, OGC regulates the supply of the relevant metabolites—malate and 2‐oxoglutarate—for OXPHOS (Figure 8A). During oxidative stress, the activity of phospholipid hydrolysis catalyzed by PLA increases significantly.^43^ As a result, the blood concentration of FAs increases in pathological conditions such as obesity and type II diabetes.^44^ In addition to the increase in free FAs, oxidative stress leads to the formation of lipid hydroperoxides and reactive aldehydes, which can modify and activate UCPs.^45^, ^46^ Under these conditions, OGC is likely to be involved in FA‐mediated proton transport (Figure 8B).

We found that 2‐oxoglutarate is a competitive inhibitor of OGC‐mediated proton leak, with an EC50 value of 60 μM (Figure 6B). Considering that human plasma, liver, and brain contain 8–12 μM,^47^ 150–300 μM,^48^ and 600–800 μM^49^ of 2‐oxoglutarate, respectively, it seems that the cavity of OGC is permanently occupied and not available to bind FAs. Yet, it has been observed that 2‐oxoglutarate levels are significantly reduced when mitochondrial function is impaired or damaged by oxidative stress, such as in diabetes^50^ and breast cancer.^51^ Under conditions that promote elevated levels of FAs and reactive aldehydes, it is plausible that OGC participates in proton transport to prevent further damage. It remains to be unraveled whether OGC alone contributes to significant proton leak in vivo. Unlike UCP1 in BAT under cold acclimation conditions, individual proteins such as OGC, ANT1, UCP2, or UCP3 may not induce a substantial amount of proton transport. However, their collective uncoupling function could lead to a beneficial and transient decrease in MMP, mitigating further oxidative damage without the need for upregulation of protein expression.

The use of mitochondrial carriers through targeted activation of their uncoupling function could be a promising approach for future cancer therapy. Cancer cells have an abnormally high MMP (~220 mV compared to 140 mV in normal cells),^2^ which is associated with increased metastasis and more invasive tumor properties.^52^, ^53^ Therefore, they take up lipophilic chemical protonophores with a higher affinity than normal cells, which results in uncoupling of the IMM through activation of several proteins of the SLC25 family and a substantial decrease in MMP, which induces apoptosis. OGC is a good candidate for targeted uncoupling because it is ubiquitously expressed in healthy tissues and therefore most likely in all cancer types (Figure 1).

In conclusion, by reconstituting OGC as the only protein in the lipid bilayer membrane, we have demonstrated its role in facilitating proton transport in the presence of FAs and DNP. ATP, OGC substrates, and PS inhibited this effect, indicating competition for the same binding site. We identified residue R90 which is involved in both uncoupling and substrate transport in OGC and may play a critical role in the conserved mechanism of uncoupling in the entire SLC25 family.

## 4 MATERIALS AND METHODS

### 4.1 Chemicals

1,2‐Dioleoyl‐*sn*‐glycero‐3‐phosphotidylcholine (DOPC, #SLCF9767), 1,2‐dioleoyl‐*sn*‐glycero‐3‐phosphoethanolamine (DOPE, #SLCB7462), cardiolipin (CL, #SLCC2621), adenosine 5′‐triphosphate (ATP, #SLBZ3783), α‐ketoglutaric acid (2‐oxoglutaric acid, #BCCC4294), phenylsuccinic acid (#MKBS0493V), l‐malic acid (#SLCD6882), N‐laurylsarcosine (#L5125), Triton X‐114 (#MKCL3391), Triton X‐100 (#STBJ5677), dithiothreitol (DTT, #BCCG5712), 2,4‐dinitrophenol (D198501), dimethylsulfoxide (DMSO, #276855), bovine serum albumin (BSA, #SLCM9403), arachidic acid (Ara, #0000145297), cis‐11‐eicosaenoic acid (20‐1, #MKCP4439), cis‐11,14‐eicosadienoic acid (20‐2, #SLCL2548), cis‐8,11,14‐trienoic acid (#SLCG0821), protease inhibitor cocktail (#P8340), bromophenol blue (#BCBF8233V), hexane (#296090), hexadecane (#296317), and isopropanol (#I9516) were purchased from Sigma‐Aldrich (Vienna, Austria). Sodium sulfate (Na_2_SO_4_, #8560.3), sodium chloride (NaCl, #9265.1), potassium chloride (KCl, #6781.3), 2‐(N‐morpholino) ethanesulfonic acid (MES, #4256.2), tris(hydroxymethyl)‐aminomethane (Tris, #4855.2), ethylenediaminetetraacetic acid (EDTA, #8043.2), ethylene glycol‐bis(β‐aminoethyl ether)‐N,N,N′,N′‐tetraacetic acid (EGTA, #8043.1), isopropyl β‐d‐1‐thiogalactopyranoside (IPTG, #2316.3), chloramphenicol (#3886.2), kanamycin (#T832.1), β‐mercaptoethanol (#4227.3), glycerine (#3783.1), ethanol (#T913.1), desoxycholic acid sodium salt (#3484.2), sodium dodecyl sulfate (SDS, #0183.3), agarose (#3810.2), and Ponceau S staining solution (#5938.2) were purchased from Carl Roth GmbH & Co. K.G. (Karlsruhe, Germany). Chloroform (#AE 54.1) was obtained from either Carl Roth GmbH & Co. K.G. (Karlsruhe, Germany) or PanReac AppliChem (UN1888, Darmstadt, Germany). We purchased arachidonic acid (AA, #10‐2004‐7) from Larodan (Solna, Sweden), n‐octylpolyoxyethylene (#1000013726) from BACHEM (Bubendorf, Switzerland), and hydroxyapatite (#130‐0420), Bio‐Beads SM‐2 (#152‐3920) and the enhanced chemiluminescence (ECL) western blotting reagent (#170‐50001) from Bio‐Rad Laboratories (Hercules, CA, USA). Dulbecco's phosphate‐buffered saline (DPBS, #14190144) was obtained from Thermo Fisher Scientific, Waltham, MA, USA, PhosSTOP™ (#59124600) from Roche Diagnostics (Mannheim, Germany), and nuclease‐free water (#7732‐18‐5) from VWR (Vienna, Austria). ^14^C‐malic acid was purchased either from Perkin Elmer (Waltham, MA, USA) (#2625350) or from Hartmann Analytic (Braunschweig, Germany) (ARC 0771). The Ultima‐Gold™ scintillation liquid (#7722011) was purchased from Perkin Elmer (Waltham, MA, USA) and Sephadex™ G‐50 (#10297028) from Cytiva Sweden AB (Uppsala, Sweden).

### 4.2 Animals and protein sample preparation

Two‐month‐old female C57BL/6 wild‐type mice used in this study were kept under standardized laboratory conditions (12:12 h light/dark cycle, room temperature (24°C), food and water ad libitum) and sacrificed by CO_2_ asphyxiation. Mouse organ and tissue samples were pooled from at least five mice to obtain enough protein. They were homogenized with a mixer mill (MM200, Retsch, Germany) in RIPA buffer (50 mM Tris, 150 mM NaCl, 1% desoxycholic acid sodium salt, 1 mM EDTA, 1% Triton X‐100, 0.1% SDS) supplemented with a protease inhibitor cocktail. After 30 min of incubation on ice, the lysates were centrifuged 2 × 10 min at 2500 ×*g*. The supernatants were collected, aliquoted, and stored frozen at −20°C.

Total protein isolation from cancer cells was performed as described in Ref.[54] In brief, the cells were washed twice in ice‐cold DPBS and centrifuged at 300 ×*g* for 5 min. Pellets were snap frozen and sonicated in RIPA buffer supplemented with a protease inhibitor cocktail and PhosSTOP™. Lysates were centrifuged 2 × 10 min at 2500 ×*g*, and the collected supernatants were aliquoted and stored at −20°C. Total protein concentrations of tissue and cancer cell samples were determined using the Pierce BCA Protein Assay Kit (#RG235622, Thermo Fisher Scientific, Waltham, MA, USA). Further steps were performed as described for protein isolation from mouse organ tissues.

### 4.3 Western blot analysis

Western blotting was adapted to the previously published protocol.^55^ In brief, total protein was separated on 15% SDS‐PAGE gels and transferred to nitrocellulose membranes. Reversible Ponceau S staining was used as a loading control (Figure S1A). After blocking the membranes in 2% BSA blocking solution at RT, they were incubated with primary antibodies against OGC (anti‐SLC25A11, sc‐515 593, #G2016, Santa Cruz Biotechnology, Dallas, TX, USA) or succinate dehydrogenase (SDHA, ab14715, #G3365497‐8, Abcam, Cambridge, UK) overnight at 4°C. Detection was performed with the UVP ChemStudio Imaging System (Analytik Jena, Jena, Germany) using horseradish peroxidase‐linked anti‐mouse (#38) and anti‐rabbit (#29) secondary antibodies (Cell Signaling Technology, Danvers, MA, USA), and the ECL western blotting reagent. All primary and secondary antibodies were diluted with 2% BSA block solution. The semi‐quantitative analysis of western blots was done using Vision Works software version 9.1 (Analytik Jena, Jena, Germany). SDHA was used as a mitochondrial marker. The values were averaged from at least three different membranes and two biological replicates. For each biological replicate, tissues from five mice were pooled together to isolate sufficient protein levels.

### 4.4 mRNA expression analysis

RNA isolation and quantitative reverse transcription (qRT–PCR) were performed as previously described.^55^ RNA was isolated using the innuSOLV RNA Reagent (Analytik Jena, Jena, Germany) according to the guanidine isothiocyanate/phenol method. Briefly, homogenized tissue samples were incubated with 1 mL of the innuSOLV RNA reagent per 100 mg. Phase separation was done by the addition of chloroform, and RNA was precipitated with isopropanol and washed with ethanol. Air‐dried RNA was dissolved in nuclease‐free water and quantified using the NanoDrop® UV–Vis Spectrophotometer (Thermo Fisher Scientific, Waltham, MA, USA).

Two μg of RNA were subjected to DNase digestion and reverse transcribed to cDNA using random hexamer primers and the High‐Capacity cDNA Reverse Transcription Kit (both Thermo Fisher Scientific, Waltham, MA, USA). RT‐qPCR was conducted on qTOWER^3^ 84 (Analytik Jena, Jena, Germany) at a 62°C annealing temperature using the Luna® Universal qPCR Master Mix (New England BioLabs GmbH, Frankfurt am Main, Germany) and primers for *Mus musculus* solute carrier family 25 member 11 (SLC25A11) sourced from the NCBI primer‐blast [NM_024211.3]. Primers targeting both murine splice variants were used: (5′–>3′ forward: GTTGTTTGAGCGCCTGACTG, reverse: CAGCTGGAAGCCGACCAT). Relative expression levels of *mOgc* were calculated by the Δ cycle threshold (Ct) to the housekeeping gene *mRpl4* (2^−[Ct(*mOgc*)−Ct(*mRpl4*)]^).

### 4.5 Recombinant protein production and purification

Cloning, purification, and reconstitution of murine OGC were adapted to the previously established protocols.^9^, ^56^ In brief, pET24a expression plasmids containing selected OGC cDNA sequences were transformed into the *E. coli* Rosetta (DE3) pLysS strain (Novagen®, Darmstadt, Germany) and selected on kanamycin (25 μg/mL) and chloramphenicol (34 μg/mL) containing plates. Successful transformants were inoculated into the growth medium containing chloramphenicol (34 μg/mL) and grown until the optical density at 600 nm reached 0.5. Protein production was then induced with 0.5 mM IPTG, and the cells were collected by centrifugation after 3 h. IBs containing the expressed protein were isolated via high‐pressure homogenization and centrifugation. The expression of mOGC in IBs was confirmed using the Western blot analysis (Figure S1C).

### 4.6 Reconstitution of OGC into liposomes

To purify and reconstitute the protein, 2 mg of IBs were solubilized in a TE/G buffer containing 2% N‐lauroylsarcosine, 1.3% Triton X‐114, 0.3% N‐octylpolyoxyethylene, 1 mM DTT, and 2 mM phenylsuccinate (PS) at pH 7.5. The lipid mixture (DOPC:DOPE:CL; 45:45:10 mol%) was hydrated overnight and added in gradually. The mixture was concentrated and dialyzed against a buffer used in the experiments (50 mM Na_2_SO_4_, 10 mM MES, 10 mM Tris, and 0.6 mM EGTA at pH 7.34). The dialysate was centrifuged and applied to a hydroxyapatite column to remove unfolded and aggregated protein fractions. Subsequently, non‐ionic detergents were removed by Bio‐Beads SM‐2. The final protein concentration was measured with the Micro BCA Protein Assay Kit (#OI191202, Thermo Fisher Scientific, Waltham, MA, USA). Protein purity was verified by SDS‐PAGE and silver staining (Figure 3A). Proteoliposomes were produced in independent batches. The following batch numbers were used for this study: OGC #5, #9, #11, #12, #15, #23, #24, and #25.

### 4.7 Generation of the OGC R90S mutant

In vitro site‐directed mutagenesis was carried out on expression plasmids containing the cDNA of mOGC as a template. The mutation was introduced with an oligonucleotide designed to alter codon Arg90 (CGC) to Ser (AGC) using a Q5™ site‐directed mutagenesis kit (#EO552, #EO554S, New England BioLabs GmbH, Frankfurt am Main, Germany). The successful introduction of mutations was confirmed by sequencing. Mutant OGC expression plasmids were transformed in the *E. coli* expression strain Rosetta(DE3)pLysS. The rest of the protocol was as described above for the OGC wild‐type, and the batch numbers used for this study were #2, #4, and #5.

### 4.8 Substrate exchange rate measurements of OGC

OGC‐containing proteoliposomes (as described above) were filled with 2 mM malate (L‐malic acid dissolved in the experimental buffer at pH 7.34) and 2 mM ^14^C labeled malate prior to extrusion. 1 mM of DTT was added to proteoliposomes after extrusion to prevent any free sulfhydryl group‐mediated aggregation. OGC facilitated transport was initiated by adding 2 mM 2‐oxoglutarate (2‐oxoglutaric acid dissolved in the experimental buffer at pH 7.34) and stopped immediately by size exclusion chromatography using SephadexTM G‐50 dextran gels at corresponding times (Figure 3B). The remaining radioactivity in proteoliposomes was measured by liquid scintillation counting (Tri‐Carb 2100TR, Perkin Elmer, Waltham, MA, USA). In the case of inhibition, 20 mM phenylsuccinate (PS, phenylsuccinic acid dissolved in the experimental buffer at pH 7.34) was added to proteoliposomes prior to extrusion to account for the random orientation of OGC in the membrane. For control measurements, the same protocol was used on empty liposomes.

### 4.9 Formation of planar bilayer membrane and measurements of the electrical parameters

Planar lipid bilayers were formed from liposomes on the tip of the dispensable plastic pipettes as previously described.^28^ Membrane formation and bilayer quality were verified by capacitance measurements (*C* = 0.715 ± 0.03 μF/cm^2^). Capacitance did not depend on the presence of the protein, AA, or other substrates, which were added to the lipid phase before membrane formation. Current–voltage (*I*–*U*) characteristics were measured by a patch‐clamp amplifier (EPC 10, HEKA Elektronik Dr. Schulze GmbH, Germany). Total membrane conductance (*G*_m_) was derived from the slope of a linear fit of the experimental data at applied voltages in the range of −50 mV to +50 mV. Lipid concentration was 1.5 mg/mL (1.875 mM) in all experiments. AA was dissolved in chloroform and added to the lipid phase before vacuuming. ATP, 2‐oxoglutaric acid, L‐malic acid, and phenylsuccinic acid were dissolved in the buffer solution (pH adjusted to 7.34) and DNP in DMSO. The amount of DMSO added to the measuring sample never exceeded 10 μL, which was previously shown not to alter the membrane conductance.^17^ The concentrations of each substrate used in the experiments are indicated in the figure legends. The relative membrane conductance was calculated according to Equation 2:

(2) $$G_{\mathrm{rel}}=\frac{G-G_{0}}{G_{1}-G_{0}}$$

where *G*_0_ is the total membrane conductance of lipid membranes reconstituted with AA, *G*_1_ is the total membrane conductance of lipid membranes reconstituted with OGC and AA, and *G* is the total specific membrane conductance of lipid membranes reconstituted with OGC, AA, and/or ATP/PS/2‐oxoglutarate/l‐malate (Figure 6).

### 4.10 Statistical analysis

Statistical analyses were performed using Sigma Plot 12.5 (Systat Software GmbH, Erkrath, Germany). Data from the electrophysiological and substrate exchange measurements are represented as mean ± standard deviation of at least three technical replicates performed on three different days. In electrophysiological measurements, each technical replicate was the mean conductance of three to ten lipid bilayer membranes formed on the same day. In substrate exchange measurements, each technical replicate stands for a new preparation of the measuring sample. Electrophysiological data were tested using the unpaired two‐tailed Student's *t*‐test. Statistical significance was defined at *p* < 0.05 (*), *p* < 0.01 (†), *p* < 0.001.

## AUTHOR CONTRIBUTIONS

**Kristina Žuna:** Investigation; writing – original draft; visualization; writing – review and editing; formal analysis. **Tatyana Tyschuk:** Investigation; writing – review and editing; formal analysis. **Taraneh Beikbaghban:** Investigation; writing – review and editing; formal analysis. **Felix Sternberg:** Investigation; formal analysis; writing – review and editing. **Jürgen Kreiter:** Writing – review and editing; formal analysis; supervision. **Elena E. Pohl:** Conceptualization; funding acquisition; writing – original draft; writing – review and editing; data curation; project administration; supervision; resources.

## FUNDING INFORMATION

This research was supported by the Austrian Science Fund (P31559‐B20 and Sonderforschungsbereich F83 to E.E.P.).

## CONFLICT OF INTEREST STATEMENT

The authors declare no conflict of interest.

## Supporting information


**Data S1.** Supporting Information.

## Data Availability

The datasets generated and/or analyzed during this study are available from the corresponding authors upon reasonable request.
