# Supplementary material for: The 2‐oxoglutarate/malate carrier extends the family of mitochondrial carriers capable of fatty acid and 2,4‐dinitrophenol‐activated proton transport
Source: Acta Physiol (Oxf). 2024 Apr 5;240(6):e14143. doi: 10.1111/apha.14143 (PMC11475482; doi:10.1111/apha.14143)
Supplement: Supplementary file 1 — Data S1. Supporting Information. [file APHA-240-e14143-s001.pdf]

Supporting information

The 2-oxoglutarate/malate carrier extends the family of mitochondrial carriers capable of fatty acid and 2,4-dinitrophenol-activated proton transport.

Kristina Žuna, Tatyana Tyschuk*, Taraneh Beikbaghban, Felix Sternberg, Jürgen Kreiter**, Elena E. Pohl***

Physiology and Biophysics, Department of Biomedical Sciences, University of Veterinary Medicine, 1210 Vienna, Austria

*Present address: Ludwig Boltzmann Institute for Traumatology, The Research Centre in Cooperation with AUVA, 1200 Vienna, Austria

**Present address: Institute of Molecular and Cellular Physiology, Stanford University School of Medicine, Stanford, CA 94305, USA

***Corresponding author. Email: elena.pohl@vetmeduni.ac.at

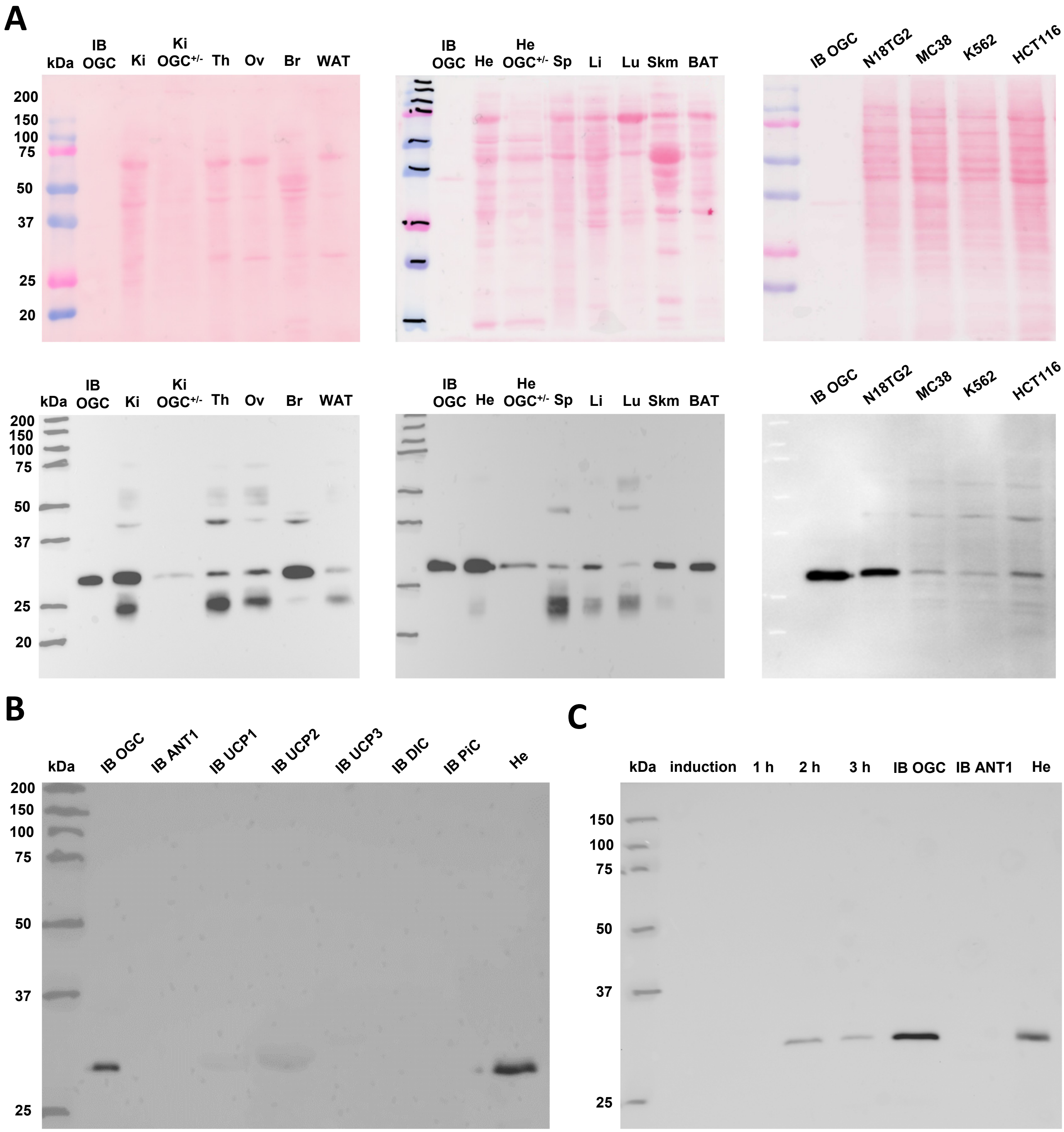

Figure S1. A. Ponceau S staining of nitrocellulose membranes (top row) and corresponding representative blots (bottom row) developed with the anti-OGC antibody and used for Western blot analysis in Figure 1. Ponceau S staining solution was applied after blotting and before incubation with the primary anti-SLC25A11 antibody. **B.** Evaluation of the specificity of the anti-SLC25A11 antibody against mOGC. Inclusion bodies (IB) with adenine nucleotide translocase 1 (ANT1), uncoupling protein 1 (UCP1), UCP2, UCP3, dicarboxylate carrier (DIC) and phosphate carrier (PIC) were tested. 40 µg of mouse heart tissue was used as a positive control for OGC. **C.** Representative Western blot of mOGC expression during protein production in *E. coli*. *m*OGC expression was induced with 0.5 mM IPTG and *E. coli* pellets were collected every hour. Production was stopped after 3 hours and the final IB were isolated. 15 µg total protein was loaded. Mouse heart tissue was used as a positive control and IB ANT1 as a negative control. Precision Plus Protein Dual Color Standard was loaded as a molecular weight marker.

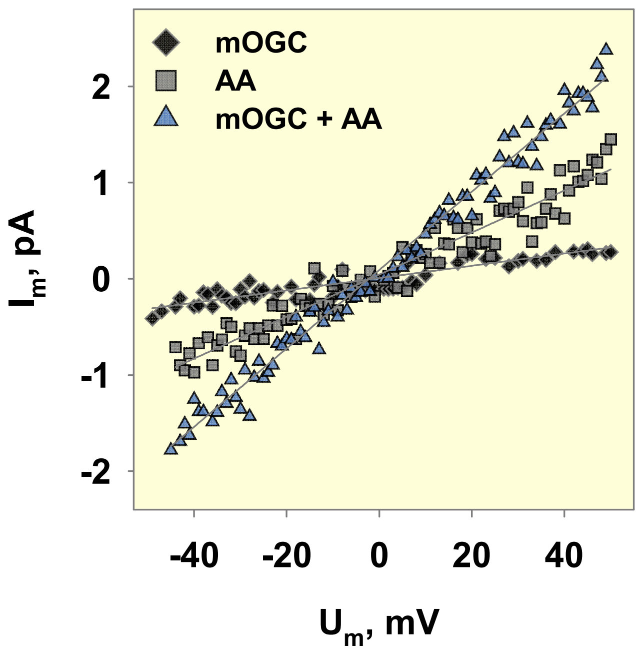

Figure S2. I/V characteristics of mOGC-mediated AA proton transport.
Representative current-voltage recordings of lipid bilayer membranes reconstituted without (black diamonds) or with AA in the absence (gray squares) or presence (blue triangles) of OGC. Other experimental conditions were similar to those described in Figure 4.

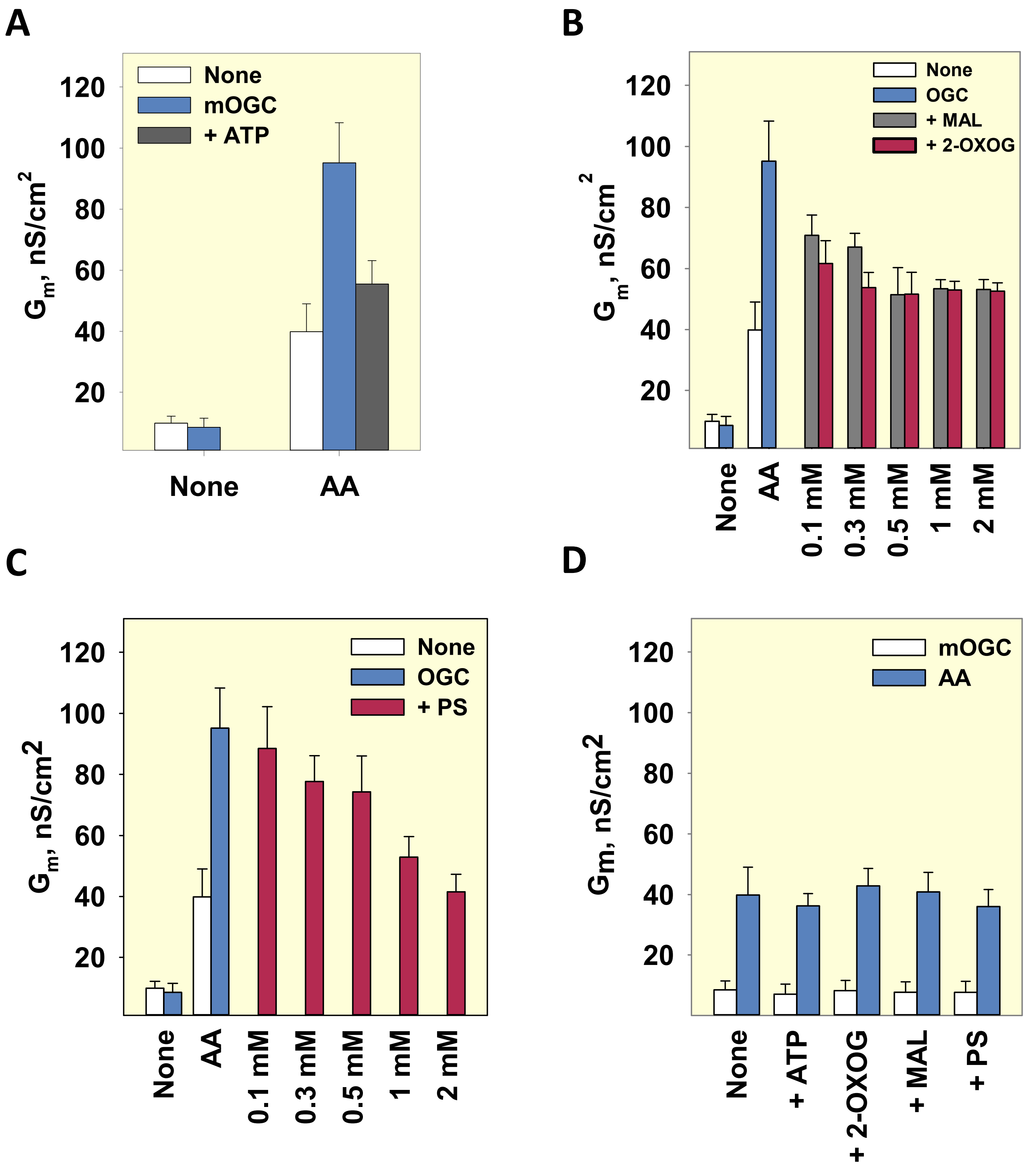

Figure S3. Total conductance (G_m) of lipid bilayer membranes reconstituted with mOGC and AA and inhibited with ATP (**A**), 2-oxoglutarate (2-OXOG) and malate (MAL, **B**) or phenylsuccinate (PS, **C**). These compounds had no effect on the G_m of lipid bilayers containing only OGC or AA (**D**). ATP, 2-OXOG and MAL were dissolved in buffer (pH = 7.34). PS was dissolved in DMSO. 2-OXOG, MAL, and PS were used at a concentration of 2 mM, and ATP was used at 4 mM, unless otherwise noted. Other experimental conditions were similar to those described in Figure 4.

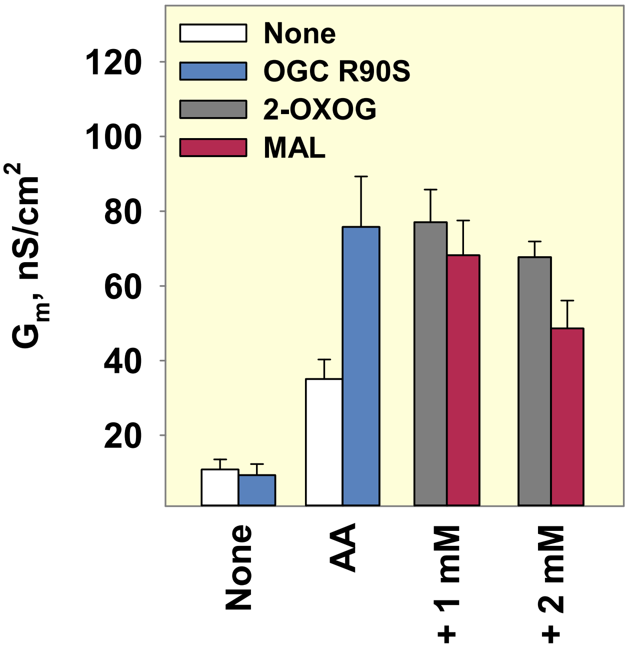

Figure S4. Increase in total membrane conductance (G_m) of mOGC-R90S in the presence of 15 mol% AA. The G_m increase was inhibited by 1 and 2 mM 2-OXOG or MAL. Other experimental conditions were similar to those described in Figure 4.

Supplementary Table 1. Substrate transport rates of SLC25 mitochondrial carriers

Protein	Protein source	Substrate exchange type	k_s (µmol min^{-1} mg^{-1})	Reference
OGC	Purified from bovine heart mitochondria	2-oxoglutarate homoexchange	6.32	(Indiveri, Palmieri et al. 1987)
PiC-B	Recombinant produced in *E. coli*	Pi-Pi homoexchange	63.6	(Fiermonte, Dolce et al. 1998)
ODC1	Purified from *S. cerevisiae* mitochondria	2-oxoglutarate/L-malate	56.4	(Palmieri, Agrimi et al. 2001)
OGC	Purified from rat brain mitochondria	2-oxoglutarate/L-malate exchange	0.64	(De Palma, Prezioso et al. 2010)
UCP2	Recombinant produced in *E. coli*	Pi/L-malate exchange	~7.5	(Vozza, Parisi et al. 2014)
OGC	Recombinant produced in *E. coli*	2-oxoglutarate/L-malate exchange	47.24	This work

OGC – 2-oxoglutarate/malate carrier
PiC-B – phosphate carrier, isoform B
ODC1 – oxodicarboxylate carrier, isoform 1
UCP2 – uncoupling protein 2
